# The Physiology Of the WEight Reduced State (POWERS) study: strategies for the analysis of biological specimens

**DOI:** 10.1038/s41366-025-01944-w

**Published:** 2026-02-05

**Authors:** Michael Rosenbaum, Kelly C. Allison, Maren R. Laughlin, Stephanie S. Kelley, Joel Nunez-Matos, Michael Rickles, Lillian Chau, Gary Wu, Payman Zamani, Kathryn J. Whyte, Rudolph L. Leibel, Matthew R. Hayes

**Affiliations:** 1 Departments of Pediatrics and Medicine, Division of Molecular Genetics, Columbia University Irving Medical Center, New York, NY USA; 2 Department of Psychiatry, Perelman School of Medicine, University of Pennsylvania, Philadelphia, PA USA; 3 Division of Diabetes, Endocrinology and Metabolic Diseases, National Institute of Diabetes and Digestive and Kidney Diseases, National Institutes of Health, Bethesda, MD USA; 4 Department of Epidemiology, University of Pittsburgh, Pittsburgh, PA USA; 5 Naomi Berrie Diabetes Center, Columbia University Irving Medical Center, New York, NY USA; 6 Division of Diabetes and Metabolic Diseases, University of Pennsylvania, Philadelphia, PA USA; 7 Division of Gastroenterology, University of Pennsylvania, Philadelphia, PA USA; 8 Department of Medicine (Cardiovascular Medicine), University of Pennsylvania, Philadelphia, PA USA; 9 Department of Pediatrics, Division of Molecular Genetics, Columbia University Irving Medical Center, New York, NY USA; 10 Columbia University, New York, NY USA; 11 AdventHealth Research Institute, Orlando, FL USA; 12 Dartmouth College, Hanover, NH USA; 13 Drexel University, Philadelphia, PA USA; 14 University of Pennsylvania, Philadelphia, PA USA; 15 Tufts University, Boston, MA USA; 16 University of North Carolina at Chapel Hill, Chapel Hill, NC USA; 17 University of Aberdeen, Glasgow, Scotland USA; 18 University of Pittsburgh, Pittsburgh, PA USA; 19 University of Florida, Gainesville, FL USA; 20 University of Kansas Medical Center, Kansas City, KS USA; 21 University of Rochester, Rochester, NY USA; 22 National Institute of Diabetes and Digestive and Kidney Diseases, Bethesda, MD USA; 23 Office of Disease Prevention, Bethesda, MD USA; 24 Office of Behavior and Social Sciences Research, Bethesda, MD USA

**Keywords:** Endocrine system and metabolic diseases

## Abstract

The Physiology Of the WEight Reduced State (POWERS) study is a multi-center clinical trial designed to understand the physiological basis for observed variability in weight regain following intentional weight loss among US adults. The primary dependent variable is weight regain over one year following a 7% or greater supervised weight loss. The overarching design and study organization, along with rationale and history of the POWERS study and outcomes measures are described in accompanying papers. This paper provides the rationale for and description of biospecimens samples that will ultimately inform on the molecular and cellular basis of the physiological variability that contributes to the regulation of energy balance in the weight reduced state. Participants will be 205 healthy adults (*n* = 205), aged 25–59 years, with body mass index (BMI) 30- ≤ 40 kg/m^2^. Biospecimens will be collected prior to weight loss (baseline, BL), immediately following weight loss via lifestyle intervention (T0), and then four (T4) and twelve (T12) months after weight loss. Blood will be collected in the fasting state and following a meal challenge designed to induce hormones related to satiety. Weight change from T0 to T12 will be the primary outcome variable. Biospecimens to be collected include plasma, serum, peripheral blood mononuclear cells (PBMCs), DNA, urine, feces, adipose and skeletal muscle tissue. All samples will be biobanked at each site. This manuscript describes the rationale for the biospecimens chosen to assess contributors to homeostatic mechanisms that drive observed variability of weight regain after weight loss.

## Introduction

The Physiology Of the WEight Reduced State (POWERS) study is a multi-site, NIH-funded discovery science initiative examining the physiological basis for the variability in weight regain among adults with obesity following intentional weight loss. Healthy adults (*n* = 205 to be enrolled), aged 25- < 60 years, body mass index [BMI] 30- < 40 kg/m^2^, will complete four intensive assessment periods. These assessments occur following 2-weeks of weight stability at initial weight (baseline, BL); immediately following a behavioral weight loss intervention resulting in at least 7% loss of initial body weight and a two-week weight stability phase (T0); four months following weight loss (T4); and 12 months following weight loss (T12). Weight stability prior to BL and T0 assessments allows for the measurement of the targets described below in a state that is not reflecting acute weight change (in either direction) at baseline, nor ongoing weight loss and acute calorie restriction at T0. Extensive collection of biological, behavioral, psychosocial, and other data relevant to each individual’s ability to maintain, as compared to regain, body weight following a behavioral weight loss intervention is performed at the four assessment timepoints. The study design, intervention, and phenotyping measures are described in accompanying papers [1–4]. This paper focuses on biospecimens (blood, urine, stool, adipose and muscle) from which potential biological substrates will be identified to define the biomechanisms of weight maintenance and weight regain in individuals with obesity who achieve a weight reduced state via a hypocaloric diet/exercise intervention.

The overarching objective of the collection of biospecimens in the POWERS trial is to enable analyses of molecular metabolic correlates of body weight status. Biospecimens collected from participants with obesity before and during the twelve months following weight loss will be examined in light of three scientific goals pertaining to the physiology of the weight reduced state and the inter-individual variability of weight maintenance: (1) To gain mechanistic insight into the many molecular inputs and outputs that likely regulate body weight. These include afferents from adipose tissue, muscle, the gastrointestinal tract, and neuroendocrine systems, as well as efferents, often from these same systems but in particular from the brain, that affect energy intake and output, as well as energy stores; (2) To identify biomarkers that are premonitory of weight regain; and (3) To generate hypotheses that can be tested in future studies of potential parameters that contribute to weight regain.

Our intent is to conduct specific analyses of banked biospecimens based on the bioenergetic, body composition, behavioral and fMRI phenotypes of participants examined at the four timepoints of the protocol, and which are described in accompanying papers [1–4]. Biospecimen analyses will be designed to generate mechanistic insights into the phenotypic changes observed. Given the speed of technological advancements that have and will continue to unfold over the years of the POWERS study, along with the need to minimize inter-assay variability in assessment of biospecimens, it is imperative to employ the most cutting-edge assays for analyses of samples when they are *all* available, which will be at the completion of the POWERS study.

The informing premise of the POWERS study is that interconnected homeostatic and hedonic/motivational mechanisms orchestrated primarily in the central nervous system act to defend against the loss of body fat by reductions in energy expenditure and increased drive to eat. The level of change in absolute adipose tissue mass, and possibly fat-free mass [5, 6], that drives these physiological and behavioral outputs at which the physiology is invoked in any individual, is determined by genetic, developmental and environmental factors [5, 6]. The relevant homeostatic (afferent) signals arise from the periphery (e.g., fat, liver, pancreas, gut, and other endocrine tissues); response (efferent) signals are generated in the brain resulting in effects on energy expenditure (via mechanisms in muscle, autonomic and neuroendocrine systems, whole body “basal metabolism”, etc.) and energy intake (food calories ingested due to hunger and hedonics).

During attempts to maintain a reduced body weight following weight loss, the postprandial milieu of satiety and adiposity signals may be viewed by the brain as insufficient relative to the pre-weight loss state of obesity. This, in turn, may lead to reduced feelings of fullness and increased frequency of hunger driving rebound hyperphagia. We are therefore proposing to measure changes in circulating appetitive hormones following weight loss as predictors of weight regain. Because these hormones influence hunger, satiety, food reward and food choice (7–9), greater changes in appetitive hormones should predict susceptibility to weight regain. These appetite hormones are not restricted to GI satiation/hunger signals [e.g., cholecystokinin (CCK), gastric inhibitory polypeptide (GIP), glucagon-like peptide-1 (GLP-1), vasoactive intestinal peptide (VIP), ghrelin, liver-enriched antimicrobial peptide-2 (LEAP2), and peptide YY (PYY)] alone, but also include humoral factors affected by changes in long-term energy status and in short-term depletion/repletion status, e.g., leptin/ghrelin and amylin/insulin, respectively. To assess both GI appetitive signals and humoral energy status signals, we are collecting serial measurements of hormone levels following an overnight fast and subsequent feeding using a mixed-meal tolerance test (MMTT) with the intent to quantify the ensuing hormonal correlates of appetitive signaling [7, 8].

Many of these signals from the gastrointestinal tract and supporting digestive organs (e.g., liver and pancreas), as well as adipose tissue and other endocrine organs (e.g., thyroid gland), engage vagal afferent pathways and/or distributed nuclei throughout the neural axis, including nuclei classically associated with homeostatic controls of energy balance and those involved in modulation of reward and cognitive function (see 7-9,12 for review). Amylin, leptin, insulin, and GLP-1 activate nuclei within the mesolimbic reward system to control the motivational aspects of ingestive behavior. Thus, the assessment of these neuroendocrine hormones will be examined in the context of behavioral measures of eating and the functional magnetic resonance imaging (fMRI) evaluations being conducted in POWERS [4].

We will also measure hormones that affect energy expenditure, which will be interpreted in the context of direct measurement of total energy expenditure using doubly labelled water (DLW) and resting energy expenditure (REE) using indirect calorimetry (2). These include thyroid hormones [T4, T3, T3, thyroid stimulating hormone (TSH)], which will be measured in the fasted state prior to the MMTT, and catecholamines measured in urine collected over 24 hours. Baseline levels of thyroid hormones predict weight loss [9, 10]; T3, T4 and TSH usually decrease with weight loss [11, 12], and larger decreases in T3 and TSH during weight loss have been shown to predict weight regain [12, 13]. In addition to these classic hormones, we will measure several metabolites (e.g., glucose, FFA, triglycerides, pyruvate, lactate) whose change over time may serve as potential predictors of weight regain.

An important and highly unique component of our analytical approach includes the assessment of neuroendocrine and VAS responses to a test meal on a given test day along with the correlation of these measures with objective temporally proximate assessment of neuronal responses (fMRI) to food [14] and the subjective measures of satiety and hunger. Since both short and long durations of sleep, as well as the timing of sleep and work activity, are associated with obesity [15, 16], coupled with the growing appreciation that time-restricted eating affects stomach-to-brain communication and adiposity measures, these all necessitate the exploration of neuroendocrine responses across the day in study participants. Free-living actigraphy assessment of sleep-wake parameters at each assessment period will be used to inform these analyses.

In all instances, prospective serial analyses of molecular/endocrine phenotypes in the same individual are intended to identify physiological processes defining energy homeostasis in the weight-reduced state [17–19] that may change over time following weight loss and correlate with weight regain. In POWERS, analyses in serum and plasma biospecimens are intended to assess regulation of energy intake (EI) and interpret VAS questionnaires of perceived measures of ingestive behavior include gastric emptying, appetite-related circulating hormones, and glucose and lipid excursions in response to a meal.

Biospecimens analyses related to energy expenditure (EE) include 24-hour urine catecholamine, gene expression, and mitochondrial function in skeletal muscle samples, which will be interpreted in light of measured muscle exercise efficiency. Further measures are planned at the end of data collection period, contingent on the availability of funds, to support muscle and adipose tissue gene expression, plasma metabolome, proteome, exosomes and exposome, and stool microbiome analyses, all of which are expected to inform interpretation of EE phenotypes.

## Biospecimens and analyses

Biospecimens and the timepoints at which they are collected are summarized in Table 1. A more complete description of the 16-day assessment done at each study timepoint is found in Table 1 of ref. [1]. Samples are collected at BL, T0, T4, and T12 with the exception of skeletal muscle obtained at T0 and T4, and adipose tissue collected at BL, T0 and T4. These two specific time points for skeletal muscle were chosen in order to provide information about an underexplored transition, namely acute adaptations in muscle and muscle mitochondrial function that take place between completion of weight loss and the first four months following, which may help to predict subsequent weight regain. This schedule will allow examination of the relationships of biospecimens to weight regain at each time point, as well as the changes in biospecimens between time points. In addition to the specific analytes described above, further analyses may range from “agnostic” measures such as metabolomics and stool microbiome, to specific analytes chosen based upon specific phenotypes at specific time points detected in our studies.

**Table 1:** Biospecimen types, timepoints, and collection.

Biospecimen	Assessment period^a^	Time during Assessment Period
Blood (serum and plasma; exosomes; PBMCs). (Buffy coat DNA at BL.)	BL, T0, T4, T12	Collected during Mixed Meal Tolerance Test (MMTT)
Urine	BL, T0, T4, T12	Day 1–15: Doubly labeled water Day 14–15: 24-h urine collection
Stool	BL, T0, T4, T12	Day 15
Adipose Tissue	BL, T0, T4	Day 16
Muscle Tissue	T0, T4	Day 16

*PBMC* peripheral blood mononuclear cells.

^a^Assessment period: BL – baseline prior to weight loss intervention, T0 – immediately following weight loss intervention, T4 – 4 months after weight loss intervention, T12 – 12 months after weight loss intervention. Each assessment period is approximately 16 days from the dosing of doubly labelled water (Day 1) through to the tissue sampling day, with an absolute window of 35 days to complete all procedures in case of scheduling issues [1].

Biospecimens are sub aliquoted and banked upon collection and will be assayed at the end of the study, except for a portion of the fresh muscle tissue used for respirometry studies [2]. Remaining aliquots, comprising approximately one third of samples, will be provided to the NIDDK Central Repository for future use by scientists inside and outside the consortium (NIDDK Central Repository Homepage (nih.gov)).

### Blood is sampled using the Mixed Meal Tolerance Test (MMTT)

All blood samples analyzed in the POWERS study are drawn as part of an MMTT, where samples drawn in the fasting state prior to the meal are used for isolation of blood components (PBMCs, exosomes) as well as proteins and small molecules, and those drawn after the meal are used for measurement of nutrients, metabolites and nutrient-responsive hormones. Serial blood collection is performed via a catheter. Fasting blood samples are drawn at the start of a MMTT during which participants, following a 12–16 h overnight fast, drink 360 mL (360 kcal, 9 g fat, 4 g CHO, 22.5 g protein) of High Protein Boost (Nestlé Health Science, Vevey, Switzerland) with 1500 mg of acetaminophen mixed into the drink to measure rate of gastric emptying^,^[20], within a 10-min period. The first two blood collection times are 10 min (-10) and 1 min (-1) before the meal is given (0) and then at 15, 30, 45, 60, 90 and 120 min after meal consumption ends.

### Plasma

We expect to measure some analytes only in the fasting (−10 min) sample at BL, T0, T4 and T12. These include molecules relevant to EI such as adiponectin, AgRP, BDNF, FGF21, and irisin; molecules related to EE such as T3, thyroxine, and TSH; and molecules related to metabolism, inflammation and nutrition such as CRP and other inflammatory markers, cholesterol, HbA1c, and myostatin. Other analytes will be measured in blood taken at all eight timepoints for each meal tolerance test. These include molecules relevant to EI such as acetaminophen (added to the high protein meal replacement and used as a marker of gastric emptying rate) [21], amylin, CCK, ghrelin, GIP, GLP-1, LEAP2, orexin A and PYY; and molecules related to metabolism, inflammation and nutrition such as free fatty acids, glucagon, glucose, glycerol, insulin, insulin C peptide and triglycerides. Appetitive hormones measured in blood taken before and after the meal are expected to change from the BL to T0 assessment periods in directions associated with calorie restriction and weight loss and correlate with changes in VAS hunger/satiety scores in the weight reduced state [4]. As weight is regained, it is hypothesized that these molecules will trend back to levels stimulated by the meal at baseline. We hope to find correlations with changes in fMRI experimental outcomes, food intake, motivations to eat, and energy expenditure per unit metabolic mass. Thyroid hormones will be measured, and it is anticipated there will be a reduction in thyroid axis activity following weight loss secondary to reduced TSH, with increased reverse T3 [11]. Standard biomarkers of insulin resistance [22] will likely be decreased following weight loss.

In addition to specific analyses listed above, the POWERS study provides an opportunity for discovery using metabolomic and exposomic approaches. The purpose of such studies is to identify endogenous and exogenous (i.e., not present except for certain environmental exposures) molecules, respectively. Metabolomics analyses are expected to be performed on fasted plasma (taken at –10 min during the mixed meal tolerance test, at BL, T0, T4 and T12) and stool (collected at BL, T0, and T4) whereas exposomic analyses will likely be performed on plasma (BL, T0, T4, and T12) and adipose tissue (BL, T0, and T4). Proteomics will also be performed in fasted plasma at all time points.

Weight maintenance, similar to usual weight and weight loss, is likely to have a genetic component [17, 23]. The anticipated sample size is insufficient by many orders of magnitude for agnostic interrogation regarding those genetic contributions to endophenotypes predicting and/or mediating weight regain. However, these endophenotypes may themselves suggest or implicate specific genes whose alleles could be examined by post hoc Sanger sequencing.

*Extracellular vesicles (EV)* are released by cells such as adipocytes from the plasma membrane (microvesicles) or from multivesicular bodies (exosomes). They are found in the plasma and contain lipids, proteins and nucleic acids. Extracellular vesicles can (1) activate receptors at specific target cells; (2) transfer molecules to the target cells and thereby change their phenotype; and (3) be used as a shuttle to carry specific molecules towards specific cells. Extracellular vesicles and their associated microparticles have been associated with body weight and numerous systems regulating EI, EE, and adiposity-related co-morbidities[24, 25]. Proteins and mRNA are examples of informative measures made in extracellular vesicles.

### Peripheral blood mononuclear cells (PBMC)

PBMCs are exposed to a range of metabolites from the diet and resulting from physiological changes in multiple tissues and are sensitive to subtle and possibly systemic transcriptomic changes. As such, they may be useful in identifying responses to weight loss interventions [26]. These cells are stored for potential future investigations examining:

- Expression of specific genes that could be implicated in explaining differences among participants in their ability or lack thereof to maintain a weight reduced state in tissues from the skeletal muscle, PBMCs, and/or adipose that might contribute to weight regain or its lack thereof will explicitly be measured to:
- Relate epigenetic status of these cells to those detected in adipose tissue and muscle, as a possible surrogate for more invasive measures of those effects in future studies;
- Relate mitochondrial gene expression patterns to mood, sleep, hunger, fullness, and ingestive behaviors (and to findings in the ex vivo skeletal muscle studies described elsewhere);
- Examine congruence of expression-related phenotypes of PBMCs or their derivatives to weight regain, or to the relevant physiological phenotypes related to that endpoint. These will be used ad hoc to generate specific cell types implicated by the tissue studies above;
- Assess changes in DNA methylation, acetylation, and open chromatin in the aggregate or for specific genes, by weight status;
- Analyze the profile of immune cells to understand if weight loss affects the immune system, and if there are immune signatures that predict weight regain.

### 24 hour acidified urine

Catecholamines will be measured in urine collected over 24 h on days 14–15 of each assessment period and will reflect the status of the sympathetic nervous system in response to weight perturbation [11].

### Abdominal subcutaneous adipose tissue

Approximately 500–1000 mg abdominal subcutaneous tissue sample is aspirated during the BL, T0, and T4 assessment periods [27]. Biopsied samples will be allocated for future examination of gene expression, cell size, exposomics and other biomarkers that affect or reflect changes in energy balance or energy stores.

### Vastus lateralis

Approximately 50–150 mg of muscle is aspirated via a Bergstrom-style needle at the T0 and T4 assessment periods [28]. Examination of metabolites, gene expression and proteomics in muscle is expected to identify the molecular mechanisms for metabolic adaptation of non-resting EE. Five to 10 mg of fresh muscle tissue is studied in a subset of participants for mitochondrial coupling and substrate metabolism using an Oroboros O2K (Oroboros, Instruments, Austria) to measure mitochondrial number (mtDNA), O_2_ consumption, nutrient consumption, ATP generation, efficiency (ATP generated)/(O_2_ or other substrate consumed), proton leak, respiratory control ratio (RCR), and reactive oxygen species generation [29]. These in vitro data provide mechanistic information to explain the clinical data obtained from in vivo studies such as cycle ergometry [2].

### Stool

Stool is collected at all assessment periods and stored for analyses of metabolomics and microbiomics [30]. While there are clear differences in both the diversity and the microbial signatures of the gut microbiome from individuals with and without obesity, it is unclear whether these differences are causal or consequential relative to body fatness. The observation that people with obesity regress towards a lean microbiome following weight loss would support the latter even though the human microbiome may be involved in propensities towards insulin resistance, inflammation, or other molecules modifying the proclivity to regain weight [31]. Supporting a causal role, studies of fecal transplants from humans, including lean and with obesity, and from mouse donors to gnotobiotic mice result in adoption of the donor somatotype by the formerly germ-free rodents. This clearly implicates the correlation of microbiome change in flora in the development of obesity, adiposity-related comorbidities, and the response to interventions designed to achieve sustained weight reduction [31, 32].

### Processing and storage

#### Blood processing

Serum is collected in SST gold top tubes at room temperature. Each vacutainer is gently inverted five times to mix the clot activator with the blood. The blood then stands upright at room temperature for 30 minutes, followed by centrifuging the sample for 10 min, 3000–3500 RPMs, at room temperature (25 °C) within 45 min of collection. The supernatant is drawn off and 0.5 ml samples are aliquoted to 2.0 ml cryovials and frozen at –80 °C.

Whole blood for hemoglobin A1c is obtained from an EDTA (lavender top) collected at MMTT −10 min. The tube is inverted 5 times and a 0.5 ml aliquot placed in a a 2 mL cryovial and placed in –80 °C.

The remaining EDTA (lavender top) sample is used for plasma isolation. After gentle inversion five times, the samples are centrifuged for 15 min, 3000–3500 RPMs, at 4 °C within 20 minutes of collection. The supernatant is drawn off and 0.5 ml samples aliquoted to 2.0 ml cryovials. All aliquots are frozen at −80 °C.

#### Plasma with protease inhibitor cocktail + DDPIV

Two EDTA (lavender top) tubes are collected. 0.1 ml of protease inhibitor and 0.05 ml of DPPIV inhibitor are kept on ice in a 0.3 ml insulin syringe and injected into the EDTA tube immediately after blood collection. After gentle inversion five times, the EDTA tubes are centrifuged for 15 min, 3000–3500 RPMs, at 4 °C beginning within 20 min of collection. The supernatant is dispenses in 0.5 ml aliquots to 2.0 ml cryovials. All aliquots are frozen at −80 °C.

Buffy coat is collected from 1 EDTA (lavender top) tube. Tubes are kept on ice upon collection. After gentle inversion five times, the samples are centrifuged for 15 min, 3000–3500 RPMs, at 4 °C beginning within 20 min of collection. Immediately after centrifugation, mononuclear cells and platelets (buffy coat) is in a whitish layer just under the plasma layer. Aspirate approximately 50–75% of the plasma without disturbing the buffy coat and aliquot 0.5 ml buffy coat sample into a 2.0 ml Cryovial. All aliquots are frozen at −80 °C.

Platelet poor plasma (PPP) is collected in sodium citrate light blue tubes at room temperature and gently inverted five times. PPP buffer is prepared at UPenn and supplied in 4 mL aliquots to both sites and frozen until use. The buffer consists of DPBS, PEG1, EDTA (RNAase-free), acetone, and phosphate buffer solution. The required number of tubes containing PPP buffer are then thawed on a benchtop. The samples are centrifuged for 20 min, 500 RCF, at 4 °C beginning within 20 min of collection. Plasma (platelet rich) is removed and mix 1:1 with PPP buffer (DBS with 2 mM EDTA and 2 μM PGE1). https://markvcid.partners.org/sites/default/files/external/protocols/MarkVCID_Plasma_Exosome_Endo_Inflam_Kit. The supernatant/PPP buffer (3.0–5.0 ml) is transferred into a 15 ml centrifuge tube. Centrifuge 15 ml tube for 20 min, 2200 RCF*, at 4 °C. Supernatant is draw off and pipetted 0.5 ml into a 2.0 ml cryovial.

4.0 ml whole blood PBMC samples are collected in a BD Vacutainer™ CPT™ Tube mononuclear cell preparation tube with sodium citrate at room temperature. Two CPT tubes are collected for each subject at each time point. Tubes are then inverted gently five times. Procedure below is for each individual CPT tube. Tubes are centrifuged) at room temperature (25–26 ˚ C) in a horizontal rotor (swing-out head) for 30 min at 1800–1900 RCF (relative centrifugal force for e.g., Eppendorf centrifuge 5810 R). After centrifugation, tubes are wiped with 70% isopropyl alcohol before being processed in a sterile hood. Mononuclear cells and platelets are in a whitish layer just under the plasma layer. Approximately half of the plasma is removed without disturbing the cell layer using a disposable sterile Pasteur pipette and discarded. The cell layer is collected from each tube with a Pasteur pipette and transferred to a sterile 15 mL conical centrifuge tube with cap. Collection of cells immediately following centrifugation yields best results. 1X PBS is added to bring the volume to 10 ml. Capped tubes are inverted five times to mix the cells and then centrifuged for 10 min at 300 RCF. In the hood the supernatant is aspirated as completely as possible without disturbing cell pellet. The cell pellet is resuspended by gently tapping tube. The wash step twice with 1XPBS is repeated twice. 1 ml of 1XPBS is added and then material is transferred to a 15 ml centrifuge tube which is tapped to resuspend cells evenly. An aliquot of 10ul is removed to count cells (Countess™ Automated Cell Counter). The remaining 990ul in the 15 ml centrifuge tube is spun for 15 minutes at 600 RCF at room temperature (25–26 ˚C). For cryopreservation, a 15 ml conical tube is wiped with 70% isopropyl alcohol after the final spin and, in the hood, the supernatant is aspirated without disturbing the pellet. The pellet is loosened by tapping the tube gently and the cells are re-suspended by adding 1.0 ml of cold Recovery™ - Cell Culture Freezing Medium. 1 ml of the re-suspended cells are then aliquoted into a 2 ml sterile cryovial tube and immediately transferred to an ice bucket. The tube is then transferred as soon as possible to NALGENE “Mr. Frosty” 1 °C freezing container and left overnight in a −80 °C freezer. On the next day the cryovial containing PBMCs is transferred to liquid nitrogen (vapor phase) storage at −100 to −125 °C.

#### Stool

The stool bucket is placed inside of a fume hood. The 2 ml cryotubes with external threads and cryoextract vial are pre-weighed and documented. When aliquoting, food debris is avoided. A wooden applicator stick is used to aliquot 100–150 mg of sample into a 2 ml cryovial, which is tightly recapped, using a wooden tongue depressor a Cryoextract vial (15 mL), is filled with stool. Using the side of the tongue depressor, excess is scraped off to not overfill, the vial is recapped and closed tightly. All samples are re-weighed. Stool is stored at −80 °C.

#### Biopsy samples: skeletal muscle

For skeletal muscle respirometry are allocated in cryovials at 10 mg each and will immediately undergo testing. Muscle samples for future assays are stored in aliquots of ~25 mg each, placed in liquid nitrogen pending storage at –80 °C after the biopsy is completed.

#### Biopsy samples: adipose

Adipose tissue samples (≥ 1 g) are processed and frozen within 30 min. Samples are washed with cold phosphate buffered saline over mesh and blotted dry. Blood stains are washed off and visible vessels are removed. Weighed specimens are aliquoted in clusters of ~250 mg into 2.0 ml cryotubes. Cryovials are placed in liquid nitrogen pending transfer to −80 °C.

All blood, isolated cell, urine, stool and tissue samples are sub-aliquoted and stored for subsequent analyses and distribution. Materials will be submitted to the NIDDK repository.

## Summary

Biospecimens are prospectively collected and banked in the POWERS study, which is focused on describing the weight-reduced state. Targeted and discovery-based analyses of these tissues will enable mechanistic insights into the anticipated changes in energy homeostasis, appetitive behaviors, and fMRI patterns in response to weight loss that will inform efforts to avoid or minimize weight regain. We anticipate that relevant signals will be detected from most or all of these materials, and we will collaborate with interested investigators in the selection and conduct of assays.
